# UNC93B1 Physically Associates with Human TLR8 and Regulates TLR8-Mediated Signaling

**DOI:** 10.1371/journal.pone.0028500

**Published:** 2011-12-02

**Authors:** Hiroki Itoh, Megumi Tatematsu, Ayako Watanabe, Katsunori Iwano, Kenji Funami, Tsukasa Seya, Misako Matsumoto

**Affiliations:** Department of Microbiology and Immunology, Hokkaido University Graduate School of Medicine, Sapporo, Japan; University of Cambridge, United Kingdom

## Abstract

Toll-like receptors (TLRs) 3, 7, 8, and 9 are localized to intracellular compartments where they encounter foreign or self nucleic acids and activate innate and adaptive immune responses. The endoplasmic reticulum (ER)-resident membrane protein, UNC93B1, is essential for intracellular trafficking and endolysosomal targeting of TLR7 and TLR9. TLR8 is phylogenetically and structurally related to TLR7 and TLR9, but little is known about its localization or function. In this study, we demonstrate that TLR8 localized to the early endosome and the ER but not to the late endosome or lysosome in human monocytes and HeLa transfectants. UNC93B1 physically associated with human TLR8, similar to TLRs 3, 7, and 9, and played a critical role in TLR8-mediated signaling. Localization analyses of TLR8 tail-truncated mutants revealed that the transmembrane domain and the Toll/interleukin-1 receptor domain were required for proper targeting of TLR8 to the early endosome. Hence, although UNC93B1 participates in intracellular trafficking and signaling for all nucleotide-sensing TLRs, the mode of regulation of TLR localization differs for each TLR.

## Introduction

The innate immune system discriminates self from non-self by expressing germ-line encoded receptors that recognize pathogen- or damage-associated molecular patterns [1]-[3]. The Toll-like receptor (TLR) family of type I transmembrane proteins were the first group of pattern recognition receptors to be identified [4]. Within this family, TLRs 3, 7, 8, and 9 recognize microbial nucleic acids and induce cytokine production, including type I interferon (IFN), and dendritic cell (DC) maturation [2]. In humans, TLR7 and TLR9 are selectively expressed in B cells and plasmacytoid DCs, while TLR3 and TLR8 are expressed in myeloid DCs [5]–[8].

TLR8 is phylogenetically and structurally related to TLR7 [9], [10]; they both recognize ssRNA and an imidazoquinoline compound [11]–[13]. In mice, TLR8 appears to be nonfunctional in most tissues and cells, except for the brain [14], [15]. Human TLR8 is expressed in myeloid cells, such as monocytes, macrophages, and myeloid DCs, and also, in regulatory T cells [6], [16], [17]. Upon stimulation with synthetic ligands, human TLR8 activates NF-κB via the adaptor protein MyD88, which leads to the induction of proinflammatory cytokines but, not type I IFN [13]. In contrast, human/mouse TLR7 strongly induces type I IFN production in response to ssRNA and an imidazoquinoline compound. The differential expression and cytokine profiles of human TLR8 compared with those of human/mouse TLR7 suggest that human TLR8 plays a distinct role in the anti-viral immune response.

Notably, these nucleotide-sensing TLRs are localized to intracellular compartments. Human TLR3 localizes to the early endosome where it recognizes exogenous dsRNA and generates signals via Toll/interleukin-1 receptor (TIR)-containing adaptor molecule-1 (TICAM-1), also named TIR domain-containing adaptor-inducing IFN-β [8], [18]–[20]. A linker region between the transmembrane domain and the TIR domain consisting of 26 amino acids determines the subcellular localization of human TLR3 [21]. In contrast, the transmembrane domain is the main determinant for intracellular localization of mouse TLR7 and TLR9 [22], [23]. The endoplasmic reticulum (ER)-resident membrane protein, UNC93B1, physically associates with TLR7 and TLR9 and delivers them to endolysosomes [24], [25]. After the trafficking of TLR7 and TLR9 from the ER to the endolysosome, their ectodomains are cleaved to generate a functional receptor [26], [27]. UNC93B1 also interacts with TLR3 [24], but its role in the intracellular trafficking of TLR3 remains undefined; however, the interaction of UNC93B1 with the TLR3, 7, and 9 transmembrane regions is essential for the signaling function of these TLRs [28], [29]. In contrast, there is little information concerning the subcellular localization and trafficking of human TLR8.

In this study, we analyzed the subcellular localization of TLR8 in human monocytes and HeLa transfectants and demonstrated that TLR8 was localized to the early endosome and the ER but not to the late endosome or lysosome. Using a series of TLR8 deletion mutants, we demonstrated that both the transmembrane and the TIR domains are required for the intracellular localization of TLR8. Furthermore, we showed that UNC93B1 physically associates with TLR8 and regulates TLR8-mediated signaling.

## Results

### Human TLR8 localizes to the early endosome and the ER in human monocytes

**Figure 1:**
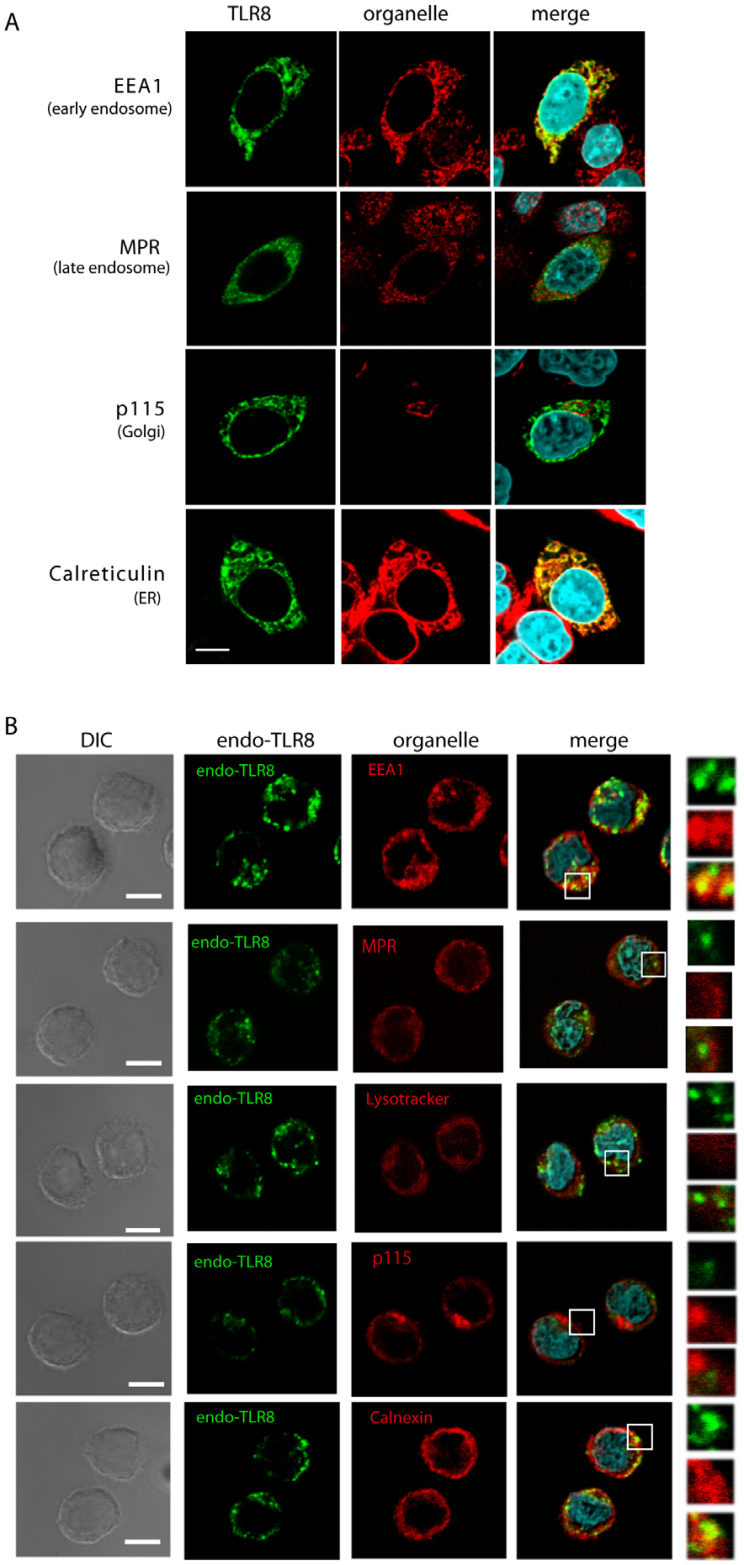
Subcellular localization of TLR8 in human monocytes and HeLa transfectants. HeLa cells transiently expressing human TLR8 (*A*) and human monocytes (*B*) were incubated with anti-FLAG mAb (*A*) or anti-TLR8 mAb (*B*) followed by an Alexa Fluor 488-conjugated secondary Ab. Organelles were stained with an anti-EEA1 pAb, anti-MPR pAb, anti-p115 pAb, anti-calreticulin pAb, or anti-calnexin pAb followed by an Alexa Fluor 568-conjugated secondary Ab. Representative confocal images are shown. Green, TLR8; red, organelle markers; blue, nuclei stained with DAPI. Scale bar: (*A*) 10 µm; (*B*) 5 µm.

Intracellular expression of TLR8 was first analyzed using a chimeric receptor composed of the extracellular domain of murine TLR4 fused with the transmembrane and cytoplasmic regions of murine TLR8 [22]. Subsequently, intact human TLR8 was shown to localize intracellularly in transfected cells [30]. However, the site of localization of TLR8 remained unknown. We therefore analyzed the subcellular localization of human TLR8 in HeLa cells transiently expressing FLAG-tagged human TLR8. When examined using confocal microscopy, TLR8-positive compartments colocalized with the early endosome antigen 1 (EEA1) and the ER marker calreticulin (Fig. 1A). Late endosome (MPR), lysosome (LAMP-1), and Golgi (p115) markers did not colocalize with TLR8 (Fig. 1A and data not shown). TLR8 is expressed in human monocytes and induces the cytokine production in response to a synthetic TLR8 ligand [13]; therefore, we next examined the subcellular localization of endogenous TLR8 in human monocytes. TLR8 was localized to the early endosome and the ER but not to the late endosome/lysosome or Golgi (Fig. 1B). Thus, the localization site of TLR8 differed from TLR7 and TLR9, both of which reside in the endolysosome and the ER.

### The TIR domain is required for endosomal localization of human TLR8

**Figure 2:**
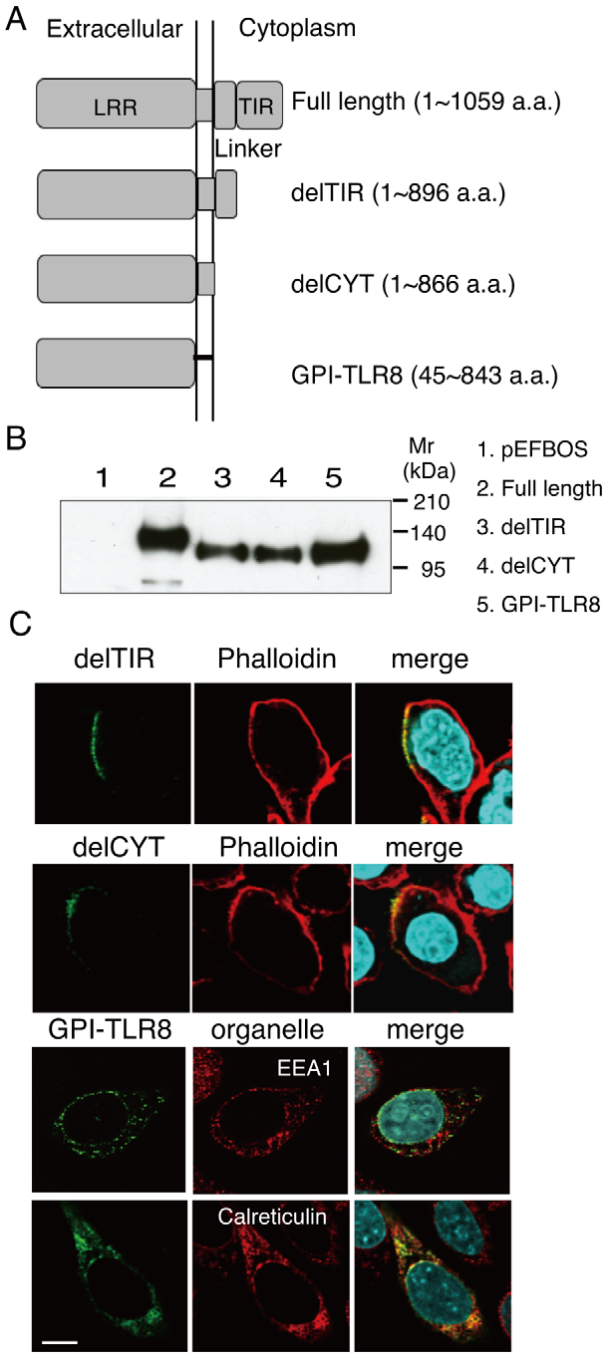
Defining the TLR domain responsible for localization. *A*, Schematic diagram of the tail-deletion constructs of hTLR8. *B*, Expression of the TLR tail-deletion constructs in HEK293FT cells. Wild-type and mutant proteins transiently expressed in HEK293FT cells were immunoprecipitated with anti-FLAG mAb, resolved using SDS-PAGE, and detected using immunoblotting with anti-FLAG mAb. Molecular weight markers are shown on the right. *C*, Immunofluorescence images of the TLR8 deletion constructs in transfected HeLa cells. The upper and middle panels show cells stained with delTIR and delCYT together with phalloidin, which labels the plasma membrane. The lower two panels show cells stained with GPI-TLR8 together with EEA1 or calreticulin. Green, TLR8 mutants; red, organelles; blue, nuclei stained with DAPI; bar, 10 µm.

To determine which region is responsible for the intracellular localization and trafficking of human TLR8, we constructed tail-truncated mutants (Fig. 2A). These included a mutant lacking the TIR domain but retaining the proximal 31 amino acids (delTIR, 1–896 a.a.), one that lacked the cytoplasmic tail (delCYT, 1–866 a.a.), and a mutant lacking the transmembrane domain and the cytoplasmic tail, which anchors the receptor to the membrane via glycosylphosphatidylinositol (GPI) (GPI-TLR8, 45–843 a.a.) (Fig. 2A). When transiently expressed in HEK293FT cells, these mutant proteins were expressed with the expected molecular weight (Fig. 2B). Immunofluorescence staining of HeLa transfectants with anti-FLAG mAb showed that both delTIR and delCYT were displayed on the cell surface (Fig. 2C), while GPI-TLR8 resided only in the ER (Fig. 2C). The lack of expression of GPI-TLR8 on the cell surface was confirmed by flow cytometric analysis with an anti-FLAG mAb (Fig. S1). These results suggest that both transmembrane and the TIR domains are required for proper targeting of human TLR8 to the early endosome.

### UNC93B1 physically associates with human TLR8

**Figure 3:**
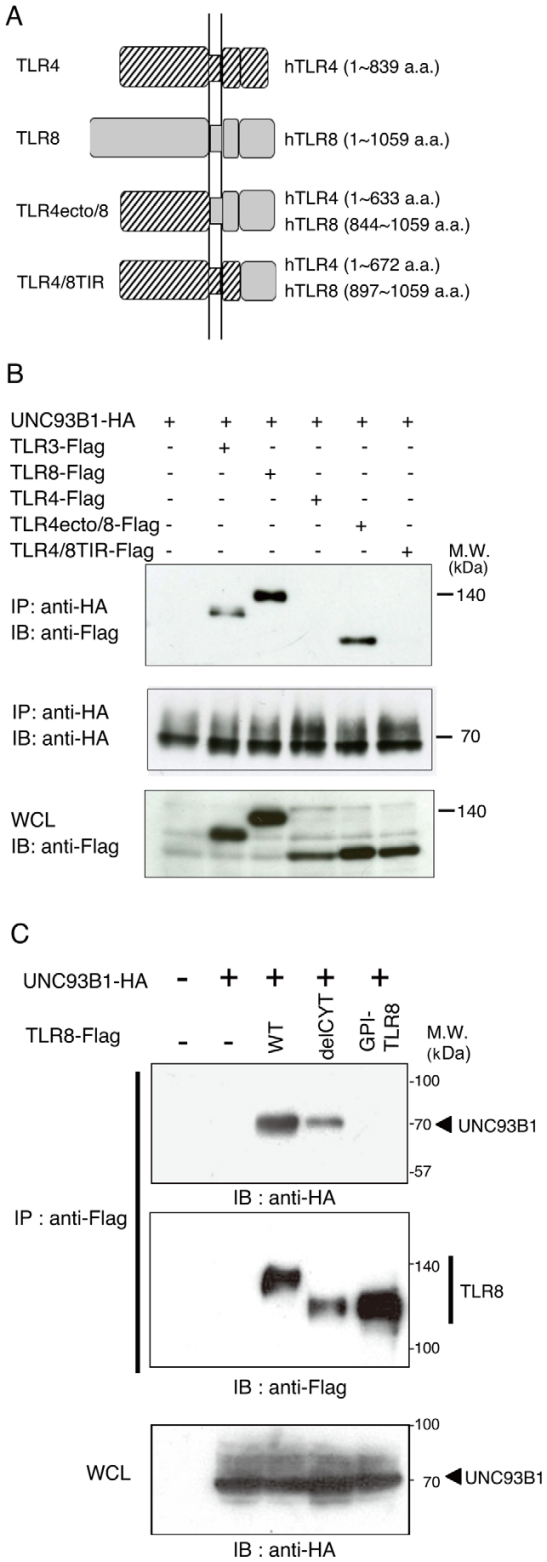
UNC93B1 physically associates with TLR8 through the transmembrane domain in HEK293FT cells. *A*, Schematic diagram of the TLR4/8 chimeric receptor constructs. *B*, *C,* HEK293FT cells were transfected with the corresponding vectors for expression of the indicated proteins. Twenty-four hours after transfection, cells were lysed in lysis buffer. The lysates were immunoprecipitated (IP) with anti-HA pAb (*B*) or anti-FLAG pAb (*C*), resolved using SDS-PAGE, and detected using immunoblotting (IB) with anti-FLAG M2 mAb or anti-HA mAb. Whole cell lysates (WCL) were subjected to immunoblotting with anti-FLAG mAb (*B*) or anti-HA mAb (*C*) to detect protein expression. Molecular weight markers are shown on the right.

The ER membrane protein UNC93B1 interacts with TLR3, TLR7, and TLR9 in the ER through the transmembrane domain, and is critical for signaling by these TLRs. Imaging analyses revealed that UNC93B1 regulates intracellular trafficking and endolysosomal targeting of TLR7 and TLR9 [25]. However, its participation in TLR8 localization and signaling remains unknown. To determine the role of UNC93B1 in TLR8 function, we constructed an additional two TLR4/8 chimeric receptors and examined physical interaction between human UNC93B1 and wild-type TLR8, TLR4/8 chimeric receptors, or tail-truncated mutants using co-immunoprecipitation analysis. Chimera TLR4ecto/8 comprised the extracellular domain of TLR4 and the transmembrane and cytoplasmic regions of TLR8, while chimera TLR4/8TIR was composed of the extracellular, transmembrane, and linker regions of TLR4 and the TIR domain of TLR8 (Fig. 3A). As shown in Figure 3B, wild-type TLR8 protein co-immunoprecipitated with UNC93B1 protein in HEK293FT cell lysates, similar to the TLR3 protein. In addition, the TLR4ecto/8 chimeric protein, but not TLR4 and TLR4/8TIR proteins, physically associated with UNC93B1, indicates that the transmembrane region of TLR8 is required for interaction with UNC93B1 (Fig. 3B). Consistent with these results, the TLR8 mutant delCYT associated with UNC93B1, while GPI-TLR8 failed to interact with UNC93B1 (Fig. 3C).

### UNC93B1 colocalizes with surface-expressed TLR8 mutants

**Figure 4:**
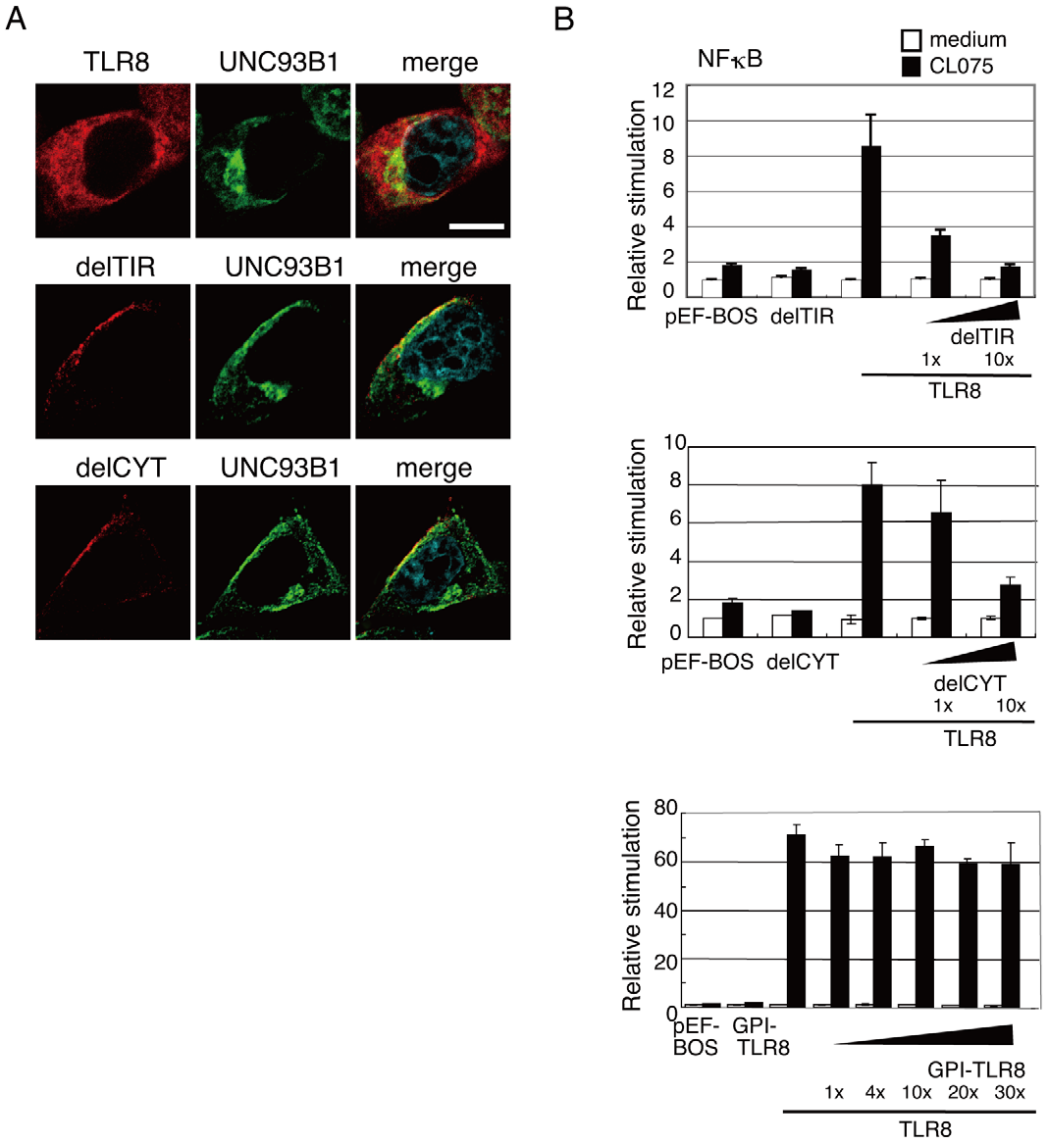
UNC93B1 colocalizes with surface-expressed TLR8 mutants. *A*, HeLa cells transiently expressing wild-type TLR8, delCYT, or delTIR were incubated with anti-FLAG mAb and anti-human UNC93B1 pAb followed by an Alexa Fluor 568-conjugated anti-mouse IgG and Alexa Fluor 488-conjugated anti-rabbit IgG. Representative confocal images are shown. Green, endogenous UNC93B1; red, TLR8; blue, nuclei stained with DAPI. Scale bar:10 µm. *B*, Surface-expressed TLR8 mutant proteins inhibited CL075-induced TLR8-mediated NF-κB activation. Luciferase activity of HEK293 cells transfected with the ELAM-promoter-luciferase reporter and expression plasmid for wild-type TLR8 together with increasing amounts of plasmid expressing delTIR (upper graph), delCYT (middle graph), or GPI-TLR8 (lower graph). Twenty-four hours after transfection, the cells were stimulated with 2.5 µg/mL of CL075 or left untreated. After 24 hours, the luciferase reporter activities were measured and expressed as the fold induction relative to the activity of unstimulated cells. Representative data from a minimum of three separate experiments are shown (mean and s.d. of triplicate assays).

Since delTIR and delCYT appeared on the cell surface (Fig. 2C), we next examined whether UNC93B1 translocates from the ER to the plasma membrane when these mutants were forcibly expressed in HeLa cells. Interestingly, confocal imaging analysis clearly demonstrated that endogenous UNC93B1 was colocalized with delTIR and delCYT at the plasma membrane, whereas wild-type TLR8 colocalized with UNC93B1 intracellularly (Fig. 4A).

Notably, delTIR and delCYT mutants performed as a dominant-negative against wild-type TLR8. As illustrated in Figure 4B, TLR8-mediated NF-κB activation induced with the TLR8 ligand, CL075 (a thiazoloquinolone derivative), was inhibited by the expression of either delTIR (Fig. 4B, upper graph) or delCYT (Fig. 4B, middle graph) in a dose-dependent manner. In contrast, the expression of GPI-TLR8 did not affect CL075-induced NF-κB activation mediated by wild-type TLR8 (Fig. 4B, lower graph). Thus, the delTIR and delCYT mutants appeared to interfere with the exit of wild-type TLR8 from the ER. Indeed, there was minimal overlay of wild-type TLR8 with EEA1 when co-expressed with these mutants (Fig. S2).

### UNC93B1 is essential for TLR8-mediated signaling

**Figure 5:**
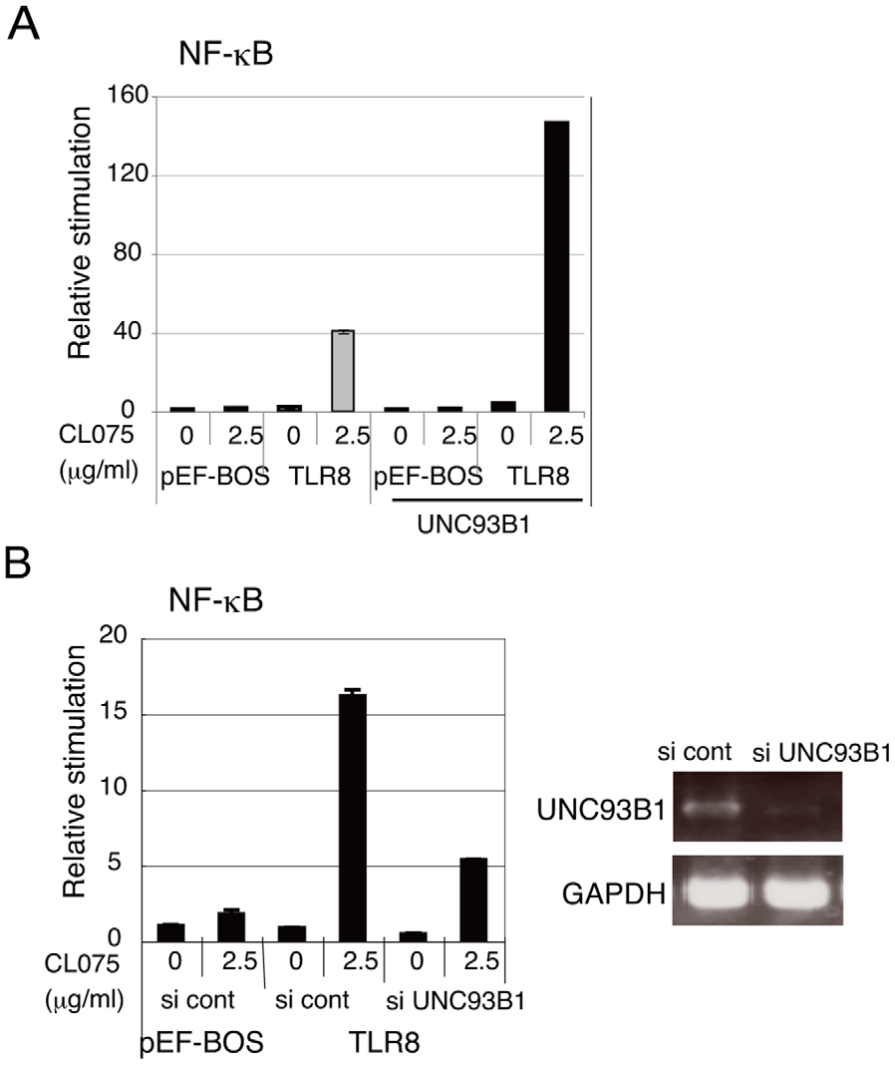
UNC93B1 is indispensable for TLR8-mediated signaling. *A*, Upregulation of TLR8-mediated NF-κB activation by co-expression with UNC93B. HEK293 cells were transfected with the indicated plasmid together with the ELAM reporter plasmid. Twenty-four hours after transfection, the cells were stimulated with CL075 or left untreated. After 6 hours, the luciferase reporter activities were measured and expressed as the fold induction relative to the activity of unstimulated cells. Representative data from three separate experiments are shown. *B*, TLR8-mediated NF-κB activation is downregulated by knockdown of UNC93B1. UNC93B1 siRNA or negative control siRNA was transfected into HEK293 cells together with the reporter plasmids and TLR8 expression plasmid. Forty-eight hours after transfection, cells were stimulated with CL075 for 6 hours and the luciferase reporter activities were measured. Data are representative of three independent experiments (mean and s.d. of triplicate assays). The expression of endogenous UNC93B1 and GAPDH mRNAs were examined using RT-PCR 48 hours after siRNA transfection (right panels).

Next, we investigated whether UNC93B1 was involved in TLR8-mediated signaling. CL075-induced TLR8-mediated NF-κB activation was greatly increased by the co-expression of UNC93B1 (Fig. 5A). Inversely, knockdown of endogenous UNC93B1 in HEK293 cells downregulated CL075-induced TLR8-mediated NF-κB activation (Fig. 5B). Thus, UNC93B1 is indispensable for TLR8 signaling.

**Figure 6:**
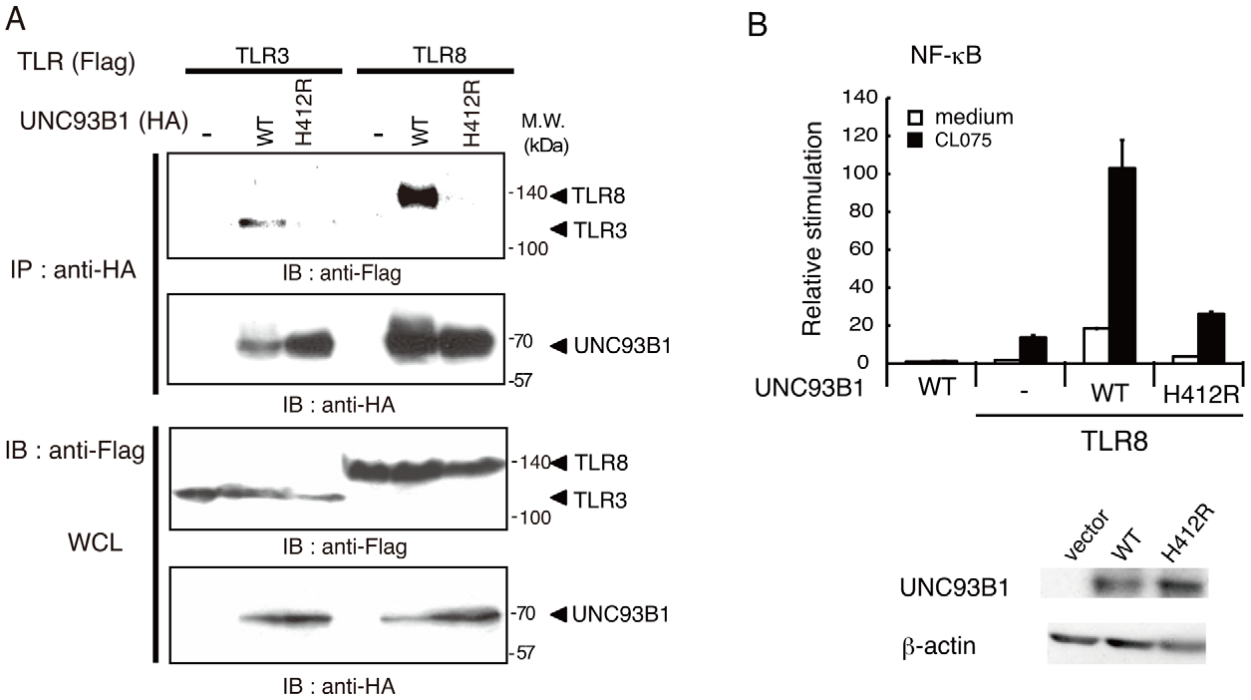
His412 is essential for the interaction of UNC93B1 with TLR8 and TLR8-mediated signaling. *A*, HEK293FT cells were transfected with the corresponding vectors for expression of the indicated proteins. The cell lysates were immunoprecipitated (IP) with anti-HA pAb, resolved by SDS-PAGE and detected by immunoblotting (IB) with anti-FLAG M2 or anti-HA mAb. Whole cell lysates (WCL) were subjected to immunoblotting with anti-FLAG or anti-HA mAb to detect protein expression. Molecular weight markers are shown on the right. *B*, HEK293 cells were transfected with the indicated plasmid together with the ELAM reporter plasmid. Cells were stimulated with CL075 or left untreated and the luciferase reporter activities were measured. Representative data from three separate experiments are shown. *Lower panels,* protein expression of wild-type and mutant UNC93B1 in HEK293 cells. β-actin blots are shown as loading controls.

Given that signaling via TLRs 3, 7, and 9 was disrupted in 3d mice, which expresses a UNC93B1 missense mutant (H412R) incapable of TLR binding [29], we constructed a human UNC93B1 mutant, hUNC93B1(H412R), and examined its ability to bind to TLR8 and mediate signaling. hUNC93B1(H412R) failed to interact with human TLR8, similar to human TLR3 (Fig. 6A). Additionally, forced expression of hUNC93B1(H412R) did not augment CL075-induced TLR8-mediated NF-κB activation (Fig. 6B), suggesting that association of human UNC93B1 with TLR8 through His412 is required for TLR8-mediated signaling as was observed with mouse UNC93B1.

## Discussion

Nucleotide-sensing TLRs are divided into two groups based on their distribution profiles in DCs. In humans, TLR3 and TLR8 are expressed in myeloid DCs, while TLR7 and TLR9 are expressed in plasmacytoid DCs [6]. Although TLR8 belongs to the TLR7/8/9 subfamily, the present study demonstrated that TLR8 possesses properties distinct from those of TLR7 and TLR9. First, TLR8, like TLR3, is localized to the early endosome but not in the late endosome/lysosome, where TLR7 and TLR9 reside. Second, although TLR8 requires UNC93B1 to exit from the ER, the TIR domain determines the endosomal targeting of TLR8.

In mouse macrophages and DCs, TLR7 and TLR9 exit the ER and travel to endolysosomes where the ectodomains of both proteins are cleaved to generate functional receptors [26], [27]. UNC93B1 controls intracellular trafficking of TLR7 and TLR9 [25], [28]. It is obvious that UNC93B1 physically interacts with TLR3, TLR7, and TLR9 in the ER through the transmembrane domain, and is critical for signaling of these TLRs in mice [24], [29]. However, prior to this study, it was unclear whether UNC93B1 was involved in TLR8-mediated signaling. We demonstrated the interaction of human UNC93B1 with human TLR8 using a co-immunoprecipitation assay and showed that the up-regulation of TLR8-mediated NF-κB activation in HEK293 cells was induced by the ectopic expression of UNC93B1 (Figs. 3 and 5). The H412R mutation within the transmembrane domain of UNC93B1 disrupted interaction between UNC93B1 and TLR8, and thus failed to increase TLR8-mediated signaling (Fig. 6). Finally, knockdown analysis revealed that UNC93B1 is indispensable for TLR8-mediated signaling (Fig. 5B).

Although UNC93B1 has been shown to deliver TLR7 and TLR9 to endolysosomes where the receptors are cleaved by proteases, this is not the case for TLR8 or TLR3. Gibbard et al. showed that the intact ectodomain of human TLR8 is necessary for dimerization of the receptor and induction of NF-κB activation in response to a synthetic ligand, CL075, in monocytes and HEK293 cells [30]. Hence, TLR8 seems to differ from TLR7 and TLR9 in its mode of ligand recognition. Interestingly, confocal analysis of TLR8 tail-truncated mutants demonstrated that endogenous UNC93B1 moved from the ER to the plasma membrane with the TLR8 mutants. In wild-type TLR8, the TIR domain controls the targeting of TLR8 to the early endosome, although UNC93B1 is required for TLR8 exit from the ER.

The nucleotide-sensing TLRs use different regulatory elements for intracellular localization. In the case of mouse TLR7 and TLR9, the transmembrane domain determines the intracellular localization of the receptor. However, Leifer et al. reported that the cytoplasmic tail of human TLR9 controls intracellular localization [31]. A 14-amino acid region in the hTLR9-TIR domain targets the Tac (human CD25)-TLR9 chimeric receptor to early endosomes. Additionally, a recent report demonstrated that bovine TLR8 localized to the ER, and that multiple regions, including ectodomain, transmembrane, linker, and TIR regions of bovine TLR8, are involved in determining the intracellular localization [32]. Thus, there may be species-specific regulatory mechanisms of intracellular localization of TLRs.

The mechanism by which TLR8 is retained in the early endosome is currently unknown. It is likely that an unidentified molecule interacts with the TIR domain of TLR8 and facilitates its trafficking to the early endosomes. As the BB loop in the TLR-TIR domain is critical for interaction with adaptor proteins, another region of the TLR8-TIR domain may participate in the association with the protein(s) regulating the receptor trafficking and intracellular localization of TLR8.

In humans, TLR7 and TLR8 recognize sequence-specific ssRNA and imidazoquinoline compounds in distinct cells and organelles, resulting in the induction of different immune responses via the same adaptor protein, MyD88. TLR7 ligands induce IFN-α production by plasmacytoid DCs, while TLR8 ligands induce proinflammatory cytokine production (e.g., TNF-α and IL-6) by myeloid DCs and monocytes [13]. This implies that TLR7 and TLR8 play distinct roles in the anti-viral immune response. Myeloid DCs express the viral RNA sensors, TLR3 and TLR8, on the endosomal membrane where they recognize virus-derived dsRNA and ssRNA, respectively. Activation of TLR3 by dsRNA results in the production of T helper 1 cytokines, such as IFN-α/β and interleukin (IL)-12p70, as well as DC maturation leading to the activation of cytotoxic T lymphocytes and natural killer (NK) cells [33]. TLR3 activation also induces TICAM-1-dependent gene expression in myeloid DCs, which mediates DC–NK reciprocal activation through cell–cell contact independently of type I IFN and IL-12 [34], whereas the key role of TLR8-mediated myeloid DC activation remains poorly understood. Detailed analyses of TLR8-mediated signaling in different cell types may give us new insight into the function of TLR8 in the anti-viral response.

## Materials and Methods

### Cell culture and reagents

HEK293 cells were maintained in Dulbecco's Modified Eagle's medium low glucose (Invitrogen) supplemented with 10% heat-inactivated FCS (BioSource Intl., Inc.) and antibiotics. HEK293FT cells were maintained in Dulbecco's Modified Eagle's medium high glucose supplemented with 0.1 mM NEAA, 10% heat-inactivated FCS and antibiotics. HeLa cells were maintained in Eagle's MEM (Nissui, Tokyo, Japan) supplemented with 1% L-glutamine and 10% heat-inactivated FCS. Human monocytes were isolated from peripheral blood mononuclear cells obtained from healthy individuals with a magnetic cell sorting system using anti-CD14-coated microbeads (Miltenyi Biotec, Gladbach, Germany). Anti-FLAG M2 monoclonal antibody (mAb), anti-HA polyclonal Ab (pAb), 4′,6-diamidine-2′-phenylindole dihydrochloride (DAPI), TRITC-labeled anti-phalloidin Ab, and saponin were purchased from Sigma-Aldrich. In addition, the following antibodies were used in this study: Alexa Fluor-conjugated secondary antibodies (Invitrogen), anti-HA mAb (Covance), anti-early endosome antigen 1 (EEA1) pAb (Affinity Bioreagents), anti-calnexin pAb and anti-calreticulin pAb (Stressgen, Victoria, Canada), anti-p115 pAb (Calbiochem, Darmstadt, Germany), anti-LAMP-1 mAb (Biolegend), anti-MPR pAb (Abcam, Cambridge, UK), anti-human TLR8 mAb (Dendritics, LYON, France) and anti-human UNC93B1 pAb (ProSci Inc., Poway, CA). Lysotracker was from Invitrogen. CL075 was from InvivoGen.

### Plasmids

Complementary DNAs for human TLR3 and TLR8 were cloned in our laboratory by RT-PCR from the mRNA of monocyte-derived immature DCs and were ligated into the cloning site of the expression vector, pEF-BOS, which was provided by Dr. S. Nagata (Kyoto University). The FLAG-tag or HA-tag was inserted into the C-terminal of pEF-BOS expression vectors for hTLR3 or hTLR8. The truncated TLR8 mutants, delTIR (1-896 a.a.) and delCYT (1-866 a.a.) were generated by PCR with Pfu Turbo DNA polymerase (STRATAGENE) using specific primers (forward primer; 5′-GACTACAAAGACGATGACGACAAGTAAGCG-3′, reverse primer for delTIR; 5′-GAAAGTTTGCGATGTGGAAAGAGACCTGTA-3′, reverse primer for delCYT; 5′-AGCCAGGGCAGCCAACATAACCATGGTGGT-3′) as described [21]. GPI-hTLR8 was constructed in the pEF-BOS expression vector by ligation of PCR products corresponding to the TLR8 ectodomain (45-843 a.a.) sequentially attached with the preprotrypsin signal sequence, HAT, and Flag at the N-terminus, and the GPI-attachment sequence from CD55 at the C-terminus. The TLR4/TLR8 chimeric receptor, TLR4ecto/8, was constructed in the expression vector pEF-BOS by ligation of PCR products corresponding to amino acids 1-633 of human TLR4 ectodomain and amino acids 844-1059 of human TLR8. Another TLR4/TLR8 chimeric receptor, TLR4/8TIR, was constructed by the ligation of PCR products corresponding to amino acids 1-672 of human TLR4 (ectodomain, TM, and linker region) and amino acids 897-1059 of the human TLR8 TIR domain. Both constructs were FLAG tagged at the C-terminus. A plasmid for human UNC93B1 (pMD2/UNC93B1) and the expression plasmid for TLR4 (pEF-BOS/TLR4) were provided by Dr. K. Miyake (The University of Tokyo). The HA-tag was inserted into the C-terminal of the pEF-BOS expression vector for human UNC93B1. The human UNC93B1 mutant, hUNC93B1(H412R), in which the arginine residue at position 412 was substituted for a histidine residue, was made by site-directed mutagenesis.

### Confocal microscopy

HeLa cells (1.0×10^5^ cells/well) were plated onto micro cover glasses (Matsunami, Tokyo, Japan) in a 24-well plate. The following day, cells were transfected with the indicated plasmids using Fugene HD (Roche Diagnostics) or Lipofectamine 2000 (Invitrogen). Twenty-four hours after transfection, cells were fixed with 3% formalin for 30 min and permeabilized with PBS containing 0.5% saponin and 1% BSA for 30 min or fixed with 4% paraformaldehyde for 30 min and permeabilized with PBS containing 0.2% Triton X-100 and 1% BSA for 15 min (for staining of endogenous UNC93B1). In the case of monocytes, cells were fixed with 4% paraformaldehyde for 15 min. For the staining of late endosome, cells were permeabilized with PBS containing 100 µg/ml of digitonin and 1% BSA for 30 minutes. Fixed cells were blocked in PBS containing 1% BSA, and were then labeled with the indicated primary Abs (2∼10 µg/ml) for 60 min at room temperature. Alexa-conjugated secondary Abs (1∶400) were used to visualize the staining of the primary Abs. After mounting with ProLong Gold with DAPI (Molecular Probes), cells were visualized at a magnification of ×63 with an LSM510 META microscope (Zeiss, Jena, Germany).

### Reporter assay

HEK293 cells (5×10^5^ cells/well) cultured in 24-well plates were transfected with the indicated plasmid together with the reporter plasmid and an internal control vector, phRL-TK (Promega), using FuGENE HD. The reporter plasmid containing the ELAM-1 promoter was constructed in our laboratory. Twenty-four hours after transfection, cells were stimulated with 2.5 µg/mL CL075. The cells were collected 6 hours after stimulation and then lysed. The *Firefly* and *Renilla* luciferase activities were determined using a dual-luciferase reporter assay kit (Promega). The *Firefly* luciferase activity was normalized with the *Renilla* luciferase activity and was expressed as the fold induction relative to the activity in unstimulated vector-transfected cells. All assays were performed in triplicate.

### RNAi

siRNA duplexes (UNC93B1:ID #s37730, negative control: catalog #AM4635) were obtained from Ambion-Applied Biosystems. HEK293 cells cultured in 24-well plates were transfected with 20 pmol of each siRNA together with the expression vector for hTLR8 (40 ng), ELAM reporter plasmid (60 ng), an internal control vector (1.5 ng) and empty vector (400 ng) using Lipofectamin 2000. Forty-eight hours after transfection, cells were washed once and then stimulated with 2.5 µg/ml CL075 for 6 hours. Knockdown of UNC93B1 was confirmed 48 hours after siRNA transfection by RT-PCR using specific primers (UNC93B1: forward primer 5′-GCCCATGATTTATTTCCTGAACCACTACC-3′ and reverse primer, 5′-GTGTGCTGAGTCCAGTCTTGTTCAG-3′, GAPDH: forward primer 5′-GAGTCAACGGATTTGGTCGT-3′ and reverse primer 5′- TTGATTTTGGAGGGATCTCG-3′). Experiments were repeated three times for confirmation of the results.

### Immunoprecipitation

HEK293FT cells (2.5×10^5^ cells/well) cultured in 12-well plates were transfected with the indicated plasmids using Lipofectamine 2000 (Invitrogen). After 24 hours, cells were washed twice with DPBS. Washed cells were lysed in 1% digitonin lysis buffer (50 mM Tris-HCl (pH 7.4), 150 mM NaCl, 5 mM EDTA, 2 mM PMSF, and a protease inhibitor cocktail) or 1% NP-40 lysis-washing buffer (50 mM Tris-HCl (pH 7.4), 150 mM NaCl, 10 mM EDTA, 1 mM PMSF, and a protease inhibitor cocktail) in Fig. 2. Lysates were clarified by centrifugation, pre-cleared with Protein G-Sepharose (GE Healthcare, Buckinghamshire, UK), and incubated with anti-FLAG mAb or anti-HA pAb. The immunoprecipitates were recovered by incubation with Protein G-Sepharose, washed three times with 0.1% digitonin washing buffer (50 mM Tris-HCl (pH 7.4), 150 mM NaCl, 5 mM EDTA, 1 mM PMSF) or 1% NP-40 lysis-washing buffer and then resuspended in denaturing buffer. Samples were analyzed by SDS-PAGE under reducing conditions followed by immunoblotting with anti-tag Abs.

## Supporting Information

Figure S1 Flow cytometric analysis of cell surface expression of GPI-hTLR8 transiently expressed in HeLa cells. HeLa cells were transfected with the empty vector or the expression plasmid for FLAG-tagged GPI-hTLR8 using Lipofectamine 2000 in 12-well plates. Twenty-four hours after transfection, cells were washed and incubated with anti-FLAG M2 mAb or mouse IgG1 in the presence of human IgG for 30 min at 4 °C in FACS buffer (DPBS containing 0.5% BSA and 0.1% sodium azide). Cells were washed twice in FACS buffer and incubated with FITC-labeled secondary antibody (American Qualex) for 30 min at 4 °C. For intracellular staining, cells were permeabilized with permeabilizing solution (BD) for 10 min at room temperature, and then stained with anti-FLAG mAb in the presence of 10% goat serum and FITC-labeled secondary Ab. Cells were analyzed using a FACS Calibur (BD). Shaded histogram: control mouse IgG 1 staining; thick line: anti-FLAG mAb staining. Inset values indicate the mean fluorescent intensities specific for the anti-FLAG mAb.
(EPS)

Figure S2 Forced expression of TLR8 mutants affects the endosomal localization of wild-type TLR8. *A*, Confocal images show HeLa cells co-expressing HA-tagged wild-type TLR8 and FLAG-tagged TLR8 mutants. Cells were fixed and stained with anti-FLAG mAb and anti-HA pAb, followed by Alexa568-labeled goat anti-mouse Ab and Alexa488-labeled goat anti-rabbit Ab. TLR8 mutants delCYT and delTIR were anchored on plasma membrane and did not merge with wild-type TLR8. Red, TLR8 mutants; green, wild-type TLR8; blue, nuclei stained with DAPI; bar, 10 µm. *B,* Cells were transfected with FLAG-tagged wild-type TLR8 alone (upper panels), together with FLAG-tagged delCYT (middle panels) or FLAG-tagged delTIR (lower panels), and stained with anti-FLAG mAb and anti-EEA1 pAb, followed by Alexa568-labeled goat anti-mouse Ab and Alexa488-labeled goat anti-rabbit Ab. Wild-type TLR8 was expressed intracellularly and colocalized with EEA1 (upper panels). When delCYT or delTIR was expressed with wild-type TLR8, colocalization between TLR8 and EEA1 was decreased (middle and lower panels). Red, TLR8; green, early endosome marker EEA1; blue, nuclei stained with DAPI; bar, 10 µm.
(EPS)

## References

[1] Medzhitov R, Janeway CA (1997). Innate immunity: the virtues of a nonclonal system of recognition.. Cell.

[2] Akira S, Uematsu S, Takeuchi O (2006). Pathogen recognition and innate immunity.. Cell.

[3] Kono H, Rock KL (2008). How dying cells alert the immune system to danger.. Nat Rev Immunol.

[4] Medzhitov R, Preston-Hurlburt P, Janeway CA (1997). A human homologue of the Drosophila Toll protein signals activation of adaptive immunity.. Nature.

[5] Muzio M, Bosisio D, Polentarutti N, D'amico G, Stoppacciaro A (2000). Differential expression and regulation of toll-like receptors (TLR) in human leukocytes: selective expression of TLR3 in dendritic cells.. J Immunol.

[6] Kadowaki M, Ho S, Antonenko S, de Waal Malefyt R, Kastelein RA (2001). Subsets of human dendritic cell precursors express different Toll-like receptors and respond to different microbial antigens.. J Exp Med.

[7] Hornung V, Rothenfusser S, Britsch S, Krug A, Jahrsdorfer B (2002). Quantitative expression of Toll-like receptor 1-10 mRNA in cellular subsets of human peripheral blood mononuclear cells and sensitivity to CpG oligodeoxynucleotides.. J Immunol.

[8] Matsumoto M, Funami K, Tanabe M, Oshiumi H, Shingai M (2003). Subcellular localization of Toll-like receptor 3 in human dendritic cells.. J Immunol.

[9] Chuang T-H, Ulevitch RJ (2000). Cloning and characterization of a sub-family of human Toll-like receptors: hTLR7, hTLR8 and hTLR9.. Eur Cytokine Netw.

[10] Du X, Poltorak A, Wei Y, Beutler B (2000). Three novel mammalian toll-like receptors: gene structure, expression, and evolution.. Eur Cytokine Netw.

[11] Heil F, Hemmi H, Hochrein H, Ampenberger F, Kirschning C (2004). Species-specific recognition of single-stranded RNA via toll-like receptor 7 and 8.. Science.

[12] Diebold SS, Kaisho T, Hemmi H, Akira S, Sousa RC (2004). Innate antivial responses by means of TLR7-mediated recognition of single-stranded RNA.. Science.

[13] Gorden KB, Gorski KS, Gibson SJ, Kedl RM, Kieper WC (2005). Synthetic TLR agonists reveal functional differences between human TLR7 and TLR8.. J Immunol.

[14] Jurk M, Heil F, Vollmer J, Schetter C, Krieg AM (2002). Human TLR7 or TLR8 independently confer responsiveness to the antiviral compound R-848.. Nat Immunol.

[15] Ma Y, Li J, Chiu I, Wang Y, Sloane JA (2006). Toll-like receptor 8 functions as a negative regulator of neurite outgrowth and inducer of neuronal apoptosis.. J Cell Biol.

[16] Peng G, Guo Z, Kiniwa Y, Voo KS, Peng W Fu (2005). Toll-like receptor 8-mediated reversal of CD4+ regulatory T cell function.. Science.

[17] Jongbloed SL, Kassianos AJ, McDonald KJ, Clark GJ, Ju X (2010). Human CD141+ (BDCA-3+) dendritic cells (DCs) represent a unique myeloid DC subset that cross-presents necrotic cell antigens.. J Exp Med.

[18] Oshiumi H, Matsumoto M, Funami K, Akazawa T, Seya T (2003). TICAM-1, an adaptor molecule that participates in Toll-like receptor 3-mediated interferon-beta induction.. Nat Immunol.

[19] Yamamoto M, Sato S, Hemmi H, Hoshino K, Kaisho T (2003). Role of adaptor TRIF in the MyD88-independent Toll-like receptor signaling pathway.. Science.

[20] Funami K, Sasai M, Ohba Y, Oshiumi H, Seya T (2007). Spatiotemporal mobilization of Toll/IL-1 receptor domain-containing adaptor molecule-1 in response to dsRNA.. J Immunol.

[21] Funami K, Matsumoto M, Oshiumi H, Akazawa T, Yamamoto A (2004). The cytoplasmic ‘linker region’ in Toll-like receptor 3 controls localization and signaling.. Int Immunol.

[22] Nishiya T, DeFranco AL (2004). Ligand-regulated chimeric receptor approach reveals distinctive subcellular localization and signaling properties of the Toll-like receptors.. J Biol Chem.

[23] Barton GM, Kagan JC, Medzhitov R (2006). Intracellular localization of Toll-like receptor 9 prevents recognition of self DNA but facilitates access to viral DNA.. Nat Immunol.

[24] Brinkmann MM, Spooner E, Hoebe K, Beutler B, Ploegh HL (2007). The interaction between the ER membrane protein UNC93B and TLR3, 7, and 9 is crucial for TLR signaling.. J Cell Biol.

[25] Kim Y-M, Brinkmann MM, Paquet M-E, Ploegh HL (2008). UNC93B1 delivers nucleotide-sensing toll-like receptors to endolysosomes.. Natur*e*.

[26] Ewald SE, Lee BL, Lau L, Wickliffe KE, Shi G-P (2008). The ectodomain of Toll-like receptor 9 is cleaved to generate a functional receptor.. Nature.

[27] Park B, Brinkmann MM, Spooner E, Lee CC, Kim YM (2008). Proteolytic cleavage in an endolysosomal compartment is required for activation of Toll-like receptor 9.. Nat Immunol.

[28] Fukui R, Saitoh S, Matsumoto F, Kozuka-Hara H, Oyama M (2009). Unc93B1 biases Toll-like receptor responses to nucleic acid in dendritic cells toward DNA- but against RNA-sensing.. J Exp Med.

[29] Tabeta K, Hoebe K, Janssen EM, Du X, Georgel P (2006). The Unc93B1 mutation 3d disrupts exogeneous antigen presentation and signaling via Toll-like receptors 3, 7 and 9.. Nat Immunol.

[30] Gibbard RJ, Morley, PJ, Gay NJ (2006). Conserved features in the extracellular domain of human Toll-like receptor 8 are essential for pH-dependent signaling.. J Biol Chem.

[31] Leifer CA, Brooks JC, Hoelzer K, Lopez J, Kennedy MN (2006). Cytoplasmic Targeting motifs control localization of Toll-like receptor 9.. J Biol Chem.

[32] Zhua J, van Drunen Littel-van den Hurka S, Brownliea R, Babiuka LA, Pottera A (2009). Multiple molecular regions confer intracellular localization of bovine Toll-like receptor 8.. Molec Immunol.

[33] Matsumoto M, Seya T (2008). TLR3: Interferon induction by double-stranded RNA including poly(I:C).. Adv Drug Del Rev.

[34] Ebihara T, Azuma M, Oshiumi H, Kasamatsu J, Iwabuchi K (2010). Identification of a polyI:C-inducible membrane protein that participates in dendritic cell-mediated natural killer cell activation.. J Exp Med.

